# Spheresomes are the main extracellular vesicles in low-grade gliomas

**DOI:** 10.1038/s41598-023-38084-y

**Published:** 2023-07-10

**Authors:** Marta Baselga, Pablo Iruzubieta, Tomás Castiella, Marta Monzón, Eva Monleón, Carmen Berga, Alberto J. Schuhmacher, Concepción Junquera

**Affiliations:** 1 Institute for Health Research Aragon (IIS Aragón), 50009 Zaragoza, Spain; 2 Department of Human Anatomy and Histology, University of Zaragoza, 50009 Zaragoza, Spain; 3 Department of Pathological Anatomy, Legal Medicine, and Toxicology, University of Zaragoza, 50009 Zaragoza, Spain; 4 Fundación Agencia Aragonesa para la Investigación y el Desarrollo (ARAID), 50018 Zaragoza, Spain

**Keywords:** Transport carrier, Cancer microenvironment, Diseases of the nervous system

## Abstract

Cancer progression and its impact on treatment response and prognosis is deeply regulated by tumour microenvironment (TME). Cancer cells are in constant communication and modulate TME through several mechanisms, including transfer of tumour-promoting cargos through extracellular vesicles (EVs) or oncogenic signal detection by primary cilia. Spheresomes are a specific EV that arise from rough endoplasmic reticulum–Golgi vesicles. They accumulate beneath cell membrane and are released to the extracellular medium through multivesicular spheres. This study describes spheresomes in low-grade gliomas using electron microscopy. We found that spheresomes are more frequent than exosomes in these tumours and can cross the blood–brain barrier. Moreover, the distinct biogenesis processes of these EVs result in unique cargo profiles, suggesting different functional roles. We also identified primary cilia in these tumours. These findings collectively contribute to our understanding of glioma progression and metastasis.

## Introduction

**Figure 1:**
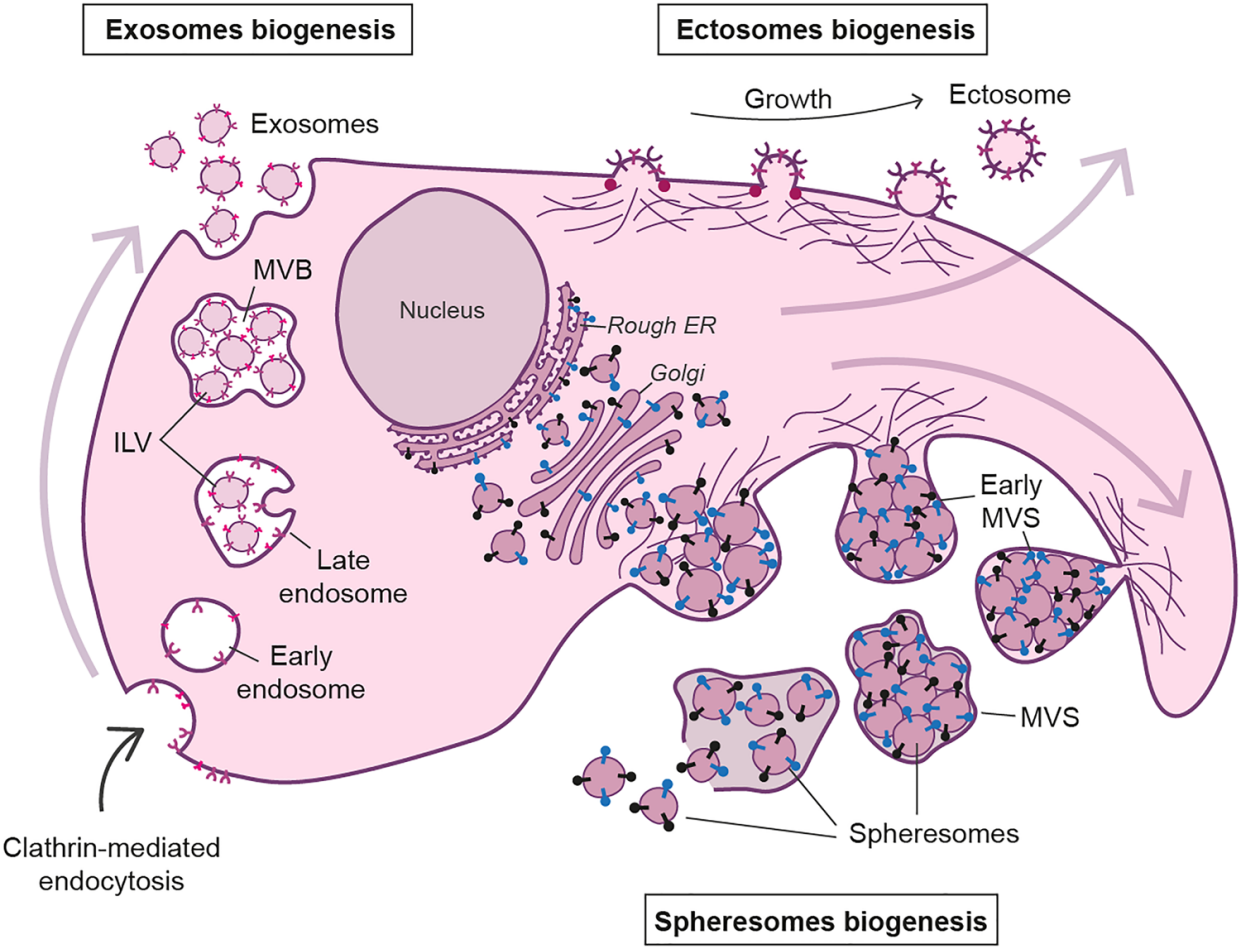
EVs biogenesis. Exosomes originate via the clathrin-mediated endocytic pathway. Cell membrane endocytosis results in the formation of early endosomes that develop late endosomes. Late endosomes’ membrane invaginates creating intraluminal vesicles (ILVs), originating multivesicular bodies (MVB). When MVB fuses with the plasma membrane, exosomes (originated from ILVs) are released. Conversly, ectosomes originate from direct budding from plasma membrane. Proteins with lipid anchors (garnet dots) accumulate and help curving the membrane as the bulge increases. Biomolecules accumulate within the bulges until they are cleaved and released as ectosomes. Spheresomes biogenesis begins via RER–Golgi-derived vesicles which aggregate beneath the cell membrane. As the number of vesicles increases, the cell membrane evaginates towards the extracellular space, originating a multivesicular sphere (MVS) that is subsequently released to the extracellular medium. MVS membrane rupture releases spherosomes. Membrane proteins (exosomes and ectosomes): pink and purples; RER-Golgi proteins (spheresomes): black and blue.

Cancer cells exchange signals both with their microenvironment and with other tumoural cells using different mechanisms. Extracellular vesicles (EVs) are raising attention in the last years as major components in intercellular communication. These lipid-bound vesicles are secreted by cells, recognize target cells and transfer biomolecules (such as proteins, lipids, mRNA, miRNA or DNA) to other cells, regulating different pathways and cellular functions. The most studied EVs are exosomes and ectosomes^1–5^. Recently, a new type of EV known as spheresome was described. Spherosomes are originated from RER–Golgi-derived vesicles that aggregate under the cell membrane. As the number of vesicles increases, the membrane evaginates into the extracellular space forming a multivesicular sphere (MVS) wich is released into this space. Lastly, MVS membrane rupture allows spherosomes to be released in the extracellular médium and contact with cells (Fig. 1)^5^. The three types of EVs present a lipid bilayer vesicle morphology and have a specific size-range of 40–160 nm (exosomes), 30–200 nm (ectosomes) and 25–100 nm (spheresomes)^5–7^. Despite their morphological similarities, the different biogenesis pathways of these EVs (Fig. 1) suggest distinct cargos and functions^1–6,8,9^.

Proteins related to EV biogenesis are upregulated in cancer cells and, consequently, EVs formation is increased compared to healthy cells^10,11^. Tumour-derived EVs carry diverse biomolecules (including proteins, miRNAs and transcripts), whose composition may vary depending on disease stage and cellular microenvironment^12,13^. These biomolecules regulate different processes including tumour progression, angiogenesis, metastasis, or drug resistance^14–17^.

Low-grade glioma are a type of neuroepithelial tumour that represents 25.2% of gliomas^18–20^. They typically exhibit good differentiation, low proliferation and low invasiveness, resulting in a favorable prognosis^21^. Despite this, low-grade gliomas tend to progress to high-grade gliomas, called anaplastic, and, eventually, to glioblastoma^22^. Therefore, a thorough comprehension of brain tumour microenvironment is crucial to understand this progression^23^.

Specifically, a single glioblastoma cell can release around 200 EVs per hour^24^, which can contact neighbour or distant cells. Locally, EVs can recruit tumoural microenvironmental cells to support tumour growth and local invasion while they can also promote tumoural metastasis through oncogenic signals transport to distant areas^25^. Glioma-derived EVs have been implicated in various tumoural events^26^. Firstly, they regulate cell viability and proliferation through signaling pathways such as PI3K-Akt, which are associated with EVs production^27^. Secondly, their cargos contain regulatory growth factors and other molecules that contribute to angiogenesis, migration, and invasion^28,29^. Thirdly, EVs are also linked to treatment resistance^30,31^. Lastly, EVs contribute to the suppression of immune mechanisms^32^. Moreover, size and composition of EVs enable them to cross barriers, including the blood brain barrier (BBB), reaching distant tissues through body fluids^33^.

General ultrastructure of low and high-grade gliomas has been scarcely described in the literature^34–40^. Electron-microscopy has been crucial to detect some features in gliomas, such as angiogenesis^23,41^, paracrystalline bodies^42^, and primary cilium^43,44^, among others. However, the presence of EVs derived from gliomas have been isolated mainly from U87 MG^45–48^ and U251^45,46,49^ cell lines^50–52^. Nevertheless, EVs have never been observed in solid human brain tumour biopsies.

Additionally, cells receive signals from the microenvironment via primary cilium^53,54^, a microtubule-based subcellular structure that protrudes from the cell and plays different roles in cellular processes, including tumoural progression^53,54^. Moser et al*.*^43^ reported that the presence of primary cilia in high-grade gliomas correlates with higher rates of tumour proliferation, showing aberrant ciliogenesis in these tumours. Conversely, Sarkisian et al.^44^ suggest the presence of the primary cilium in glioblastomas. Moreover, recent compelling studies indicate that primary cilium also emits signals through the release of EVs^23,55–57^.

EVs are involved in tumour biology, including malignization of distant cells. In addition, they show a potential role in the non-invasive diagnosis of brain tumours. To our knowledge this work provides the first evidence of spheresomes genesis and activity in low-grade human gliomas using electron microscopy. Furthermore, primary cilia biogenesis and its relation with EVs are also depicted.

## Methods

A more detailed version of the Methods can be found as Supplementary Information.

### Human samples

Human biopsies were obtained from surgical resections collected in the Department of Pathology at the University Clinic Hospital of Zaragoza, Spain. Biopsy samples from 5 patients fulfilling histological criteria of low-grade glioma were used in this study. Patients were all males of Spanish origin with a mean age of 63 (± 6.9) years. All procedures were approved by the Human Research Ethics Committee (Comité Ético de Investigación Clínica de Aragón, CEICA) from the Instituto Aragonés de Ciencias de la Salud (IACS) (permit number: PI16/0324). The experiments were performed in accordance with relevant guidelines and regulations. Informed consent was obtained from all participants in the study.

### Histological and immunohistochemistry analyses

Samples were processed according to standard histological procedures and stainining, including hematoxylin and eosin (H&E). For immunohistochemical analysis tissue sections of 2 μm were deparaffinised and antigen retrieval with citrate buffer (pH 6, DAKO S2031) was performed. Endogenous peroxidase activity was blocked using peroxidase blocking reagent (DAKO, S2001) for 10 min. Slides were incubated with polyclonal rabbit anti-GFAP (1:100, DAKO, Z0334, Glostrup, Denmark), and mouse monoclonal anti-Ki-67 (1:100, DAKO, MIB-1, Glostrup, Denmark) on 2 μm-thick formalin-fixed paraffin-embedded sections using DAKO EnVision^®^ method. The corresponding secondary antibody (Rabbit/Mouse Labelled Polymer EnVision-HRP, DAKO K5007). 3,3′ diaminobenzidine was used as chromogen. Sections were counterstained with Mayer’s haematoxylin and mounted using Eukitt (03989 Sigma-Aldrich; St. Louis, MO, USA). Digital images were obtained using Olympus BX1 microscope.

### Immunofluorescence microscopy

2 μm-thick formalin-fixed paraffin-embedded sections were deparaffinised, rehydrated and permeabilised (0.1% Triton X-100 in PBS for 8 min). Antigen retrieval was performed with Tris-buffered saline (TBS, pH 9 at 96 °C for 20 min). Slides were incubated with monoclonal mouse anti-Acetylated-tubulin (1:4000, Sigma Aldrich, T7451; St. Louis, MO, USA) or polyclonal rabbit anti-Pericentrin (1:100, Abcam, ab4448; Cambridge, UK) in a dark humidified chamber. The appropriate secondary antibody: donkey anti-mouse IgG Alexa Fluor 594 (1:1000, ThermoFisher, R37115; Waltham, MA, USA) or donkey anti-rabbit IgG Alexa Fluor 488 (1:1000, ThermoFisher, A-21206; Waltham, MA, USA) were applyied. Sections were washed with PBS, counterstained with DAPI (1 μg/mL, Sigma-Aldrich) and mounted with fluorescence mounting medium (DAKO, S3023). Samples were visualised with a fluorescence microscope (Olympus BX1 with DP70 Digital Camera System) and analysed with DP Controller Software and FIJI ImageJ software^58^.

### Transmission electron microscopy (TEM)

Tumour samples (about 1–1.5 mm^3^) were fixed with 2.5% glutaraldehyde and 2% paraformaldehyde overnight at RT, washed in 0.1 M phosphate buffer for 5 min, post-fixed with 2% osmium, rinsed, dehydrated in graded acetone (30%, 50%, 70% with 2% uranyl acetate, 90%, 100%), cleared in propylene oxide and embedded in araldite (Durcupan, Fluka AG; Buchs SG, Switzerland). Semi-thin Sections (1.5 μm) were cut with a diamond knife, lightly stained with 1% toluidine blue and examined by light microscopy (Olympus BX51 microscope, Olympus Imaging Corporation; Tokyo, Japan). Later, ultrathin (0.05 μm) sections were cut, collected on Formvar-coated single-slot grids counterstained with 1% uranyl acetate and Reynold’s lead citrate. The sections were examined under a FEI Tecnai G2 Spirit TEM. The images were captured with Advanced Microscopy Techniques, using a Corp. Charge-Coupled Device imaging system (CCD from Danvers, MA, USA). The number of MVS and MVB was counted in micrographs of 64 cells containing one or both of these vesicular structures. Within each type of MV the number of vesicles was also counted. In addition, the average diameter of MVS (number of MVS assessed, *n* = 30), MVB (*n* = 5) and of the vesicles within them (spheresomes *n* = 100; ILV or exosomes *n* = 50) was calculated using FIJI ImageJ software^58^.

## Results

### Histological and immunohistochemical features of low-grade glioma

**Figure 2:**
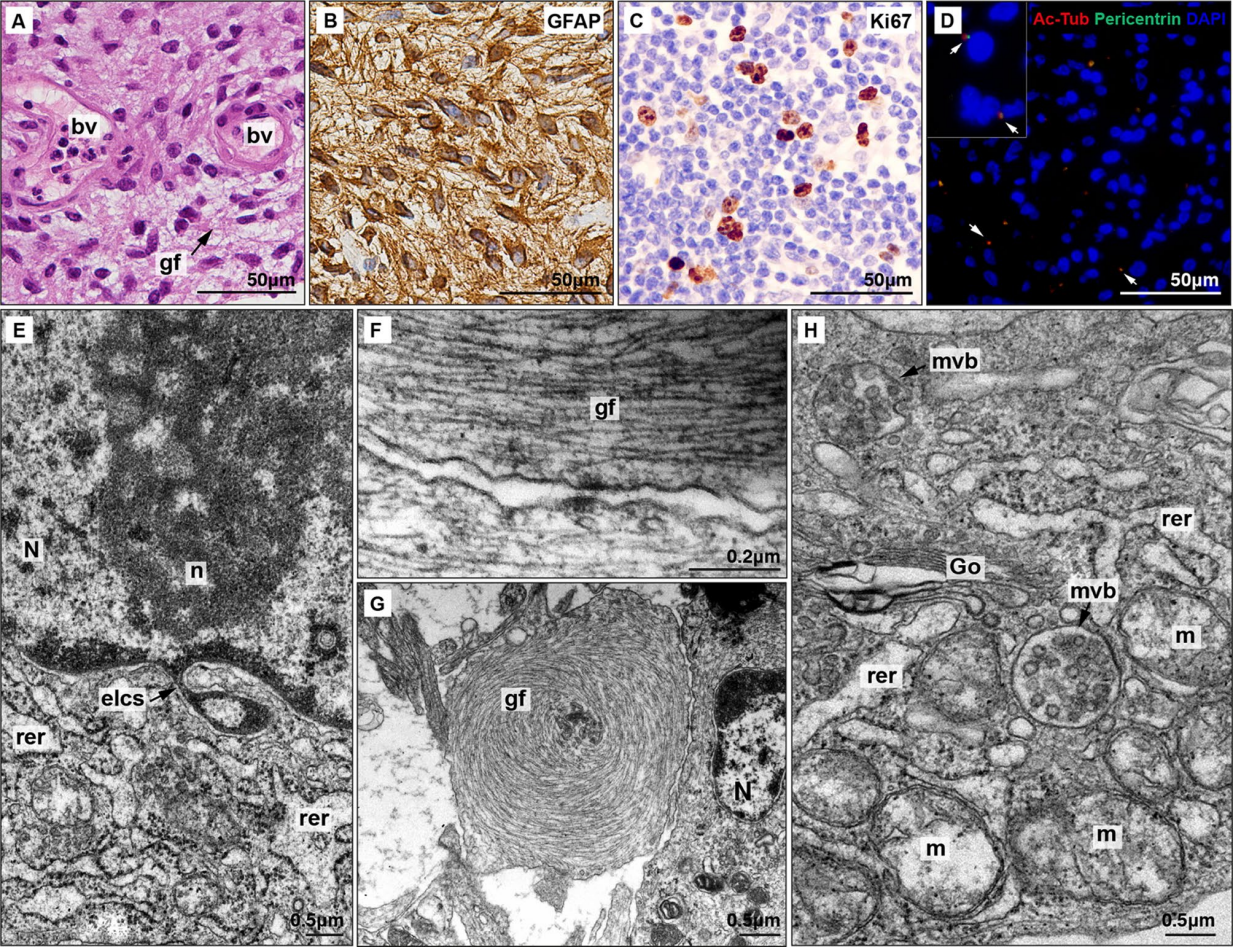
Histopathological and ultrastructural analysis of low-grade glioma. (**A**) HE staining shows increased cellularity with predominant gliofibrillar (gf) component. Blood vessels (bv) show non-hyperplasic endothelial cells. (**B**) GFAP and (**C**) Ki67 immunostaings. (**D**) Immunofluorescence; acetylated β tubulin (red) is a marker of axoneme while pericentrin (green) is present in basal body. Colocalisation of both markers show clearly the presence of primary cilia (arrows). (**E**) Electron micrograph of a nuclear envelope-limited chromatin sheets (ELCS) isolating heterochromatin (N: nucleus, n: nucleoli, rer: rugous endoplasmic reticulum). (**F**) Electron micrograph of abundant parallel gliofilaments (gf) along an astrocytic prolongation. (**G**) Electron micrograph of abnormal disposition of gliofilaments acquired a characteristic concentric shape. (**H**) Electron micrograph of multivesicular bodies (mvb) present in glioma astrocytes (Go: Golgi apparatus, m: mitochondria).

Conventional H&E study of the low-grade gliomas revealed numerous fusiform cells with a predominant fibrillar component and significant vascularisation composed by normal endothelial cells (Fig. 2A). GFAP staining confirmed an astroglial composition (Fig. 2B) while Ki67 immunostaining showed a moderate rate of mitosis (Fig. 2C). These findings confirm the diagnosis of low-grade glioma, likely a diffuse astrocytoma (WHO grade 2). Primary cilia presence was identified by co-location of acetylated beta tubulin (staining axoneme microtubules) and pericentrin (specific of centrioles and basal bodies) (Fig. 2D).

### Ultrastructural features of low-grade glioma

Tumour cells of low-grade glioma exhibit mild nuclear pleomorphism with oval nuclei that have smooth contours and a narrow band of marginal chromatin. No conspicuous nucleoli were observed, but some tumoural cells displayed nuclear envelope-limited chromatin sheets (ELCS) (Fig. 2E), which are fine nuclear envelope prolongations containing chromatin that have been previously described in other tumours. The cytoplasm of these cells contains abundant granular endoplasmic reticulum related to Golgi dyctiosomes, polyribosomes and mitochondria. Cancer cells established small contacts through cytoplasmic prolongations, composed by parallel bundles of gliofilaments (Fig. 2F). This bundles acquire sometimes an abnormal concentric disposition (Fig. 2G). Moreover, multivesicular bodies (MVB) containing intraluminal vesicles (ILV)/exosomes and MVS containing spheresomes were frequently observed (Fig. 2H). Other types of EVs (i.e. ectosomes or microvesicles) were not observed.

### Morphometry of EVs in low-grade gliomas

**Figure 3:**
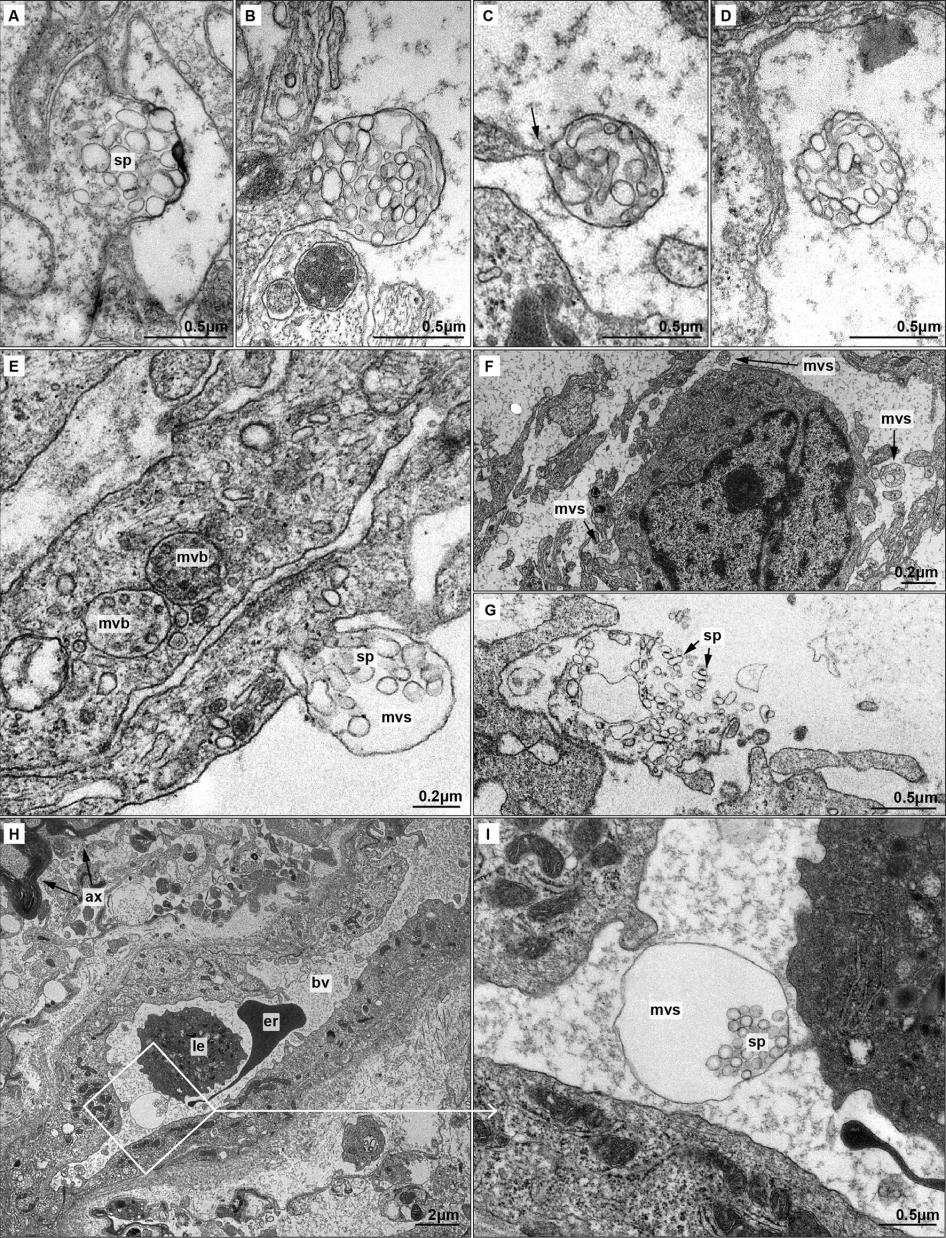
Biogenesis and local and distant migration of MVSs (**A**–**D**) Biogenesis of MVS. (**A**) Spheresomes (sp) aggregate in a specific region of the cell membrane. Cell membrane begins to evaginate pushed by spheresomes. (**B**) A membrane evagination originates the multivesicular sphere (mvs), fulfilled by spheresomes. (**C**) The cell releases the MVS by narrowing the cell membrane (arrow). MVSs frequently appear along cytoplasmatic prolongations. (**D**) MVSs are released into the extracellular medium. (**E**–**G**) Short distance migration of MVSs and spheresomes. (**E**) Comparison of exosomes-loaded multivesicular bodies (mvb) and early MVS. Differences in the size of both multivesicular structure and inside vesicles are shown. (**F**) Panoramic view of MVSs surrounding a tumoural astrocyte. (**G**) Rupture of MVS membrane releases spheresomes (**H**–**I**). Long distance migration of MVSs and spheresomes. (**H**) Small blood vessel (bv) and nearby myelin-ensheathed axons (ax). An erythrocyte (er) and a leukocyte (le) are observed in the lumen of the vessel. (**I**) Detail from (**H**) showing an MVS with a dilated membrane in the lumen of a vessel. A small extension of the endothelial cell seems to guide the MVS.

In the present study a total on 49 multivesicular spheres (MVSs) and 14 multivesicular bodies (MVBs) were observed. Comparing the frequency of EVs, we observed 3.5 times more MVSs than MVBs. Each cut section of MVSs contained 35.8 ± 16.5 spheresomes on average, while section of multivesicular bodies harbored 16 ± 5.5 exosomes. MVS showed an approximate size of 0.7 ± 0.3 μm [0.6–1.4 μm], larger than MVBs [0.4 ± 0.1 μm; 0.3–0.6 μm). Accordingly, spheresomes were also larger than exosomes (Fig. 3E): spheresomes measured 115 ± 30 nm in average [50–175 nm], while exosomes diameter were approximately 40 ± 5 nm in average [25–55 nm].

### Biogenesis of multivesicular spheres

MVSs biogenesis steps were analysed using transmission electron microscopy (TEM). Spheresomes accumulated beneath the cell membrane, protruding to the extracellular medium and originating MVS when released from the cell through cell membrane cleavage (Fig. 3A–C). Shortly after cleavage of the MVS from the membrane, the spheresomes are retained and transported packed in the MVS (Fig. 3D) until their release.

### Migration of MVSs

MVSs released from the tumoural glioma cells can transport cargos both locally and distantly. Locally, MVSs reach neighboring cells in the tumour microenvironment, frequently through prolongations from one or more of these nearby cells (Fig. 3F). Spherosomes are contained in MVSs through the extracellular space and released upon rupture of the MVS membrane (Fig. 3G).

Furthermore, MVSs might also reach distant tissues, mainly through blood vessels, as evidenced by the presence of these structures in vessels lumen (Fig. 3H,I).

### Presence and biogenesis of primary cilia

**Figure 4:**
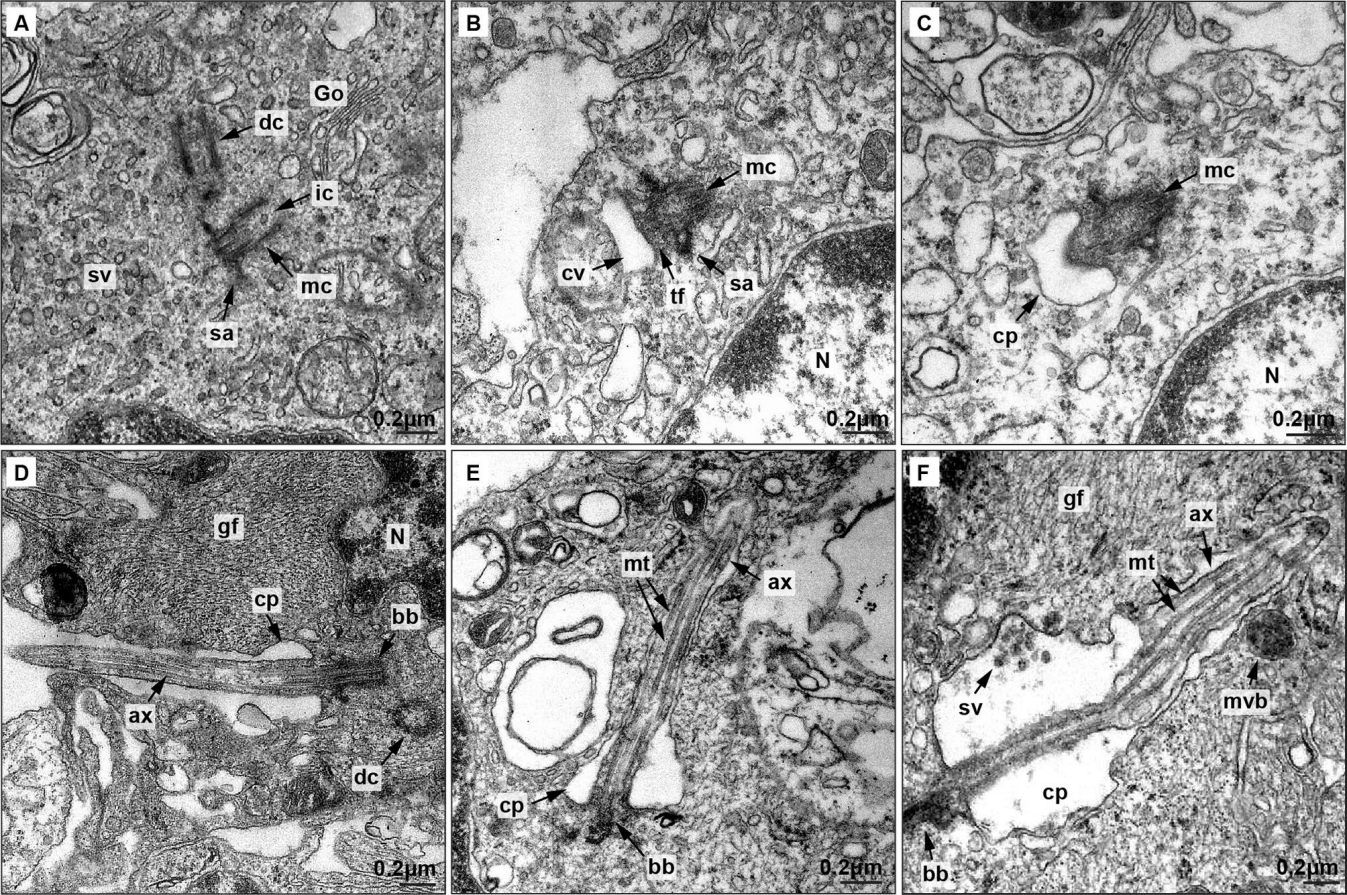
Electron microscopy analysis of primary cilia biogenesis in low-grade glioma astrocytes.(**A**–**D**) Electron microscopy of primary cilia biogenesis. (**A**) Activated mother centriole (mc) showing subdistal appendages (sa), intracentriolar cargo (ic), and abundant small vesicles (sv) in the distal pole originated in Golgi dictyosomes (Go). The daughter centriole (dc) is also visible. B) Vesicles fuse and form the ciliary vesicle (cv) anchored to mother centriole (mc) by transition fibers (tf). (**C**) Axoneme starts to grow protruding in the ciliary vesicle (cv). (**D**) Once the ciliary vesicle binds the cell membrane, cilia axoneme projects to extracellular medium. Ciliary pocket (cp) ang gliofilaments (gf) are also shown. (**E**) Ultrastructure of primary cilia in low-grade glioma astrocytes. Axoneme is composed by a microtubule cytoskeleton. (**F**) Extracellular small vesicles and multivesicular body (mvb) are close to the ciliary pocket membrane. Nucleus (N), microtubules (mt), ciliary pocket (cp), basal body (bb), axoneme (ax), gliofilaments (gf) and daughter centriole (dc) are also observed.

Primary cilia were found in low-grade gliomas, and electron microscopy was used to track ciliogenesis process in these tumoural cells. Ciliogenesis begins with the activation of one of the centrioles, the so-called mother centriole (Fig. 4A). Centriole activation is marked by the formation of subdistal appendages, cargo traffic into the centriole, and accumulation of Golgi-derived vesicles near the distal pole. These vesicles fuse to form a big ciliary vesicle (which will form the ciliary membrane) that anchors to the mother centriole through transition fibers. Then, 9 + 0 primary cilia axoneme starts to grow protruding in the ciliary vesicle (Fig. 4B–C). Finally, ciliary vesicle binds the plasma membrane and the full-length axoneme is exposed to the extracellular medium (Fig. 4D).

As depicted in Fig. 4E–F, primary cilia show structural integrity and is usually located in the perinuclear region. Notably, Fig. 4F shows the presence of small extracellular vesicles located in a region of the ciliary pocket membrane with an apparent clathrin–coated pit.

## Discussion

**Figure 5:**
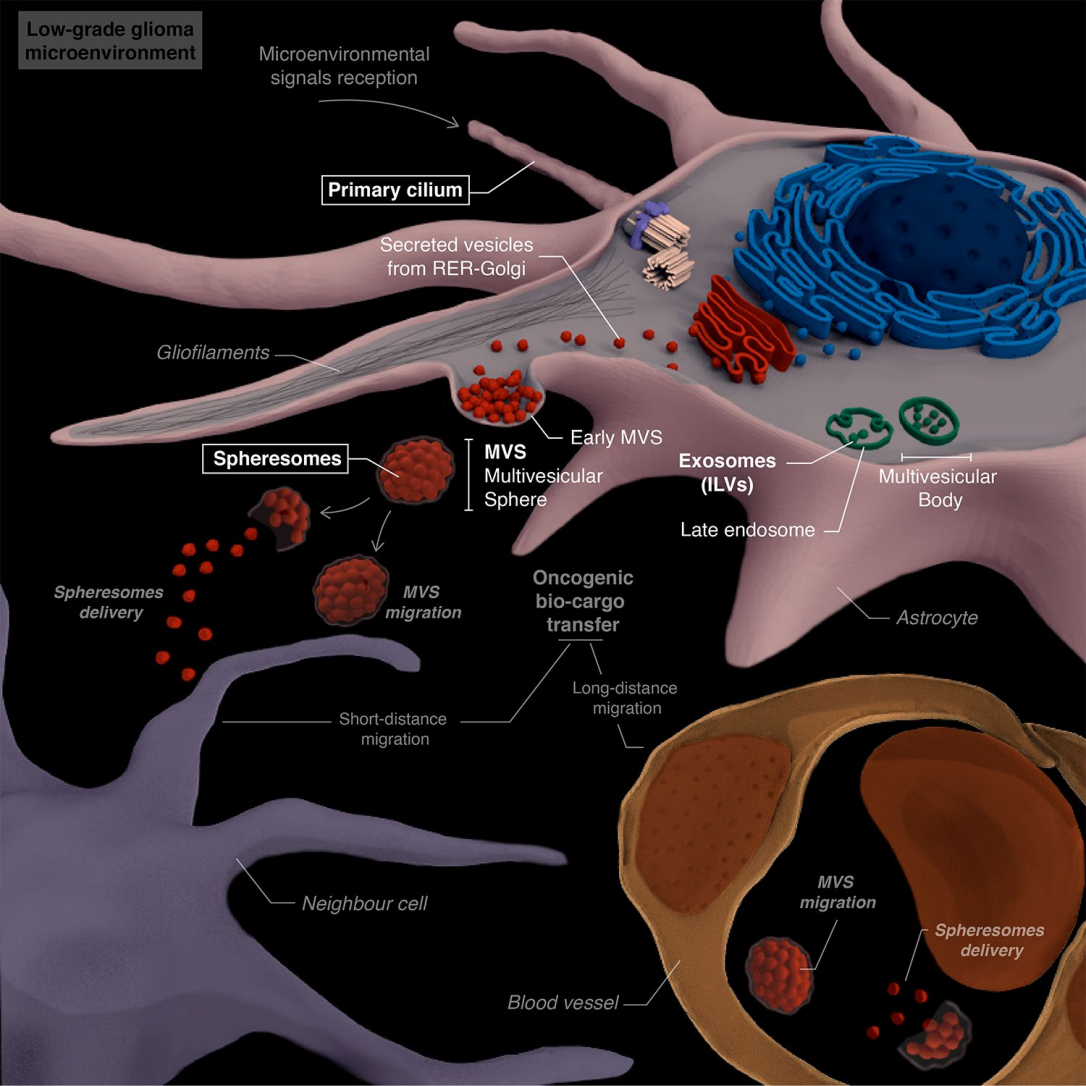
Graphical overview of the biogenesis and role of spheresomes in astrocytomas. Created with Scienfy®.

How glioma cells influence tumour microenvironment (TME) to promote progression has raised important attention^22^. Communication between tumoural cells and TME can occur through different pathways^25^, including tumour-derived EVs^59–61^. Our findings reveal that glioma tumoural cells frequently produce EVs, including multivesicular spheres (MVSs) and spheresomes, in human low-grade gliomas (Fig. 5).

Exosomes are the best-studied type of EV^62^, while spheresomes have often been misidentified as other structures, such as endocytic vesicles^52^ or exosomes^63^. However, proper study of the localisation and features of MVSs and spheresomes in various contexts, particularly in tumours, can provide insight into their functions and their potential use as biomarkers or therapeutic targets.

Analysing the distinct features of the different EVs may be crucial to understand the biology of tumours. Exosomes, ectosomes, and spheresomes share a lipid bilayer, similar morphology, and size range, being difficult to differentiate from each other^5–7^. Their biogenesis, however, is completely different^1–6,8,9^. While exosomes are generated through the endocytic pathway by budding from membrane fusion of multivesicular bodies with the cell membrane; ectosomes carry cytosolic proteins synthetised by cytosolic polyribosomes^64^. In contrast, MVSs gradually accumulate spheresomes, which are vesicles from the RER-Golgi pathway^5^. As a result, each type of EVs regulates the intercellular transport of proteins synthesised via specific pathways. Proteins synthesised by free polyribosomes accumulate as cargo in ectosomes and exosomes, while proteins synthesised in the RER–Golgi pathway would be transported by spheresomes. Therefore, the protein composition of EVs is type-specific, suggesting that spheresomes may play a unique role different from exosomes or ectosomes.

In this work we have demonstrated the coexistence of exosomes and spheresomes in human low-grade gliomas. These findings have been previously identified in other tumoural types, like gastrointestinal stromal tumours (GISTs)^5^. Our observations reveal the presence of multivesicular bodies containing exosomes in the cytoplasm of tumoural cells, which are heading towards the cell surface, as well as MVSs surrounding extracellular environment and in blood vessels lumen. In other studies regarding EVs in gliomas, MVSs were often misidentified as groups of endocytic vesicles^52^. In human biopsies, it is difficult to establish whether all tumour cells produce MVBs and MVSs or only a subset of them. However, we have been able to establish that both forms can coexist in the same tumour. Further research is needed to elucidate what specific signals regulate EVs production. Moreover, it would be important to investigate if these EVs are produced at a specific stage of the tumour progression or continuously throughout the tumour´s biology.

Tumour-derived EVs play remarkable roles in facilitating tumoural invasion and metastasis by modulating distant tissues^65–67^. In central nervous system, EVs can cross the BBB and travel through the bloodstream^68^. Research on EV cargo from glioblastoma patients has revealed different molecules that can become significant diagnostic and prognostic markers^69^. We have observed the presence of spheresomes within tumour vessels that allow MVSs to migrate to distant organs or tissues. This finding could be of great interest for early non-invasive diagnosis of these tumours. Additionally, exosomes and exosome-mimics have been used as drug delivery carriers in glioblastoma models, showing potential therapeutic applications^70–72^.

Primary cilium plays different roles in tumoural development^53,54^. The genesis of certain tumours is related to alterations in specific ciliary membrane proteins that transmit abnormal proliferation signals, including Sonic hedgehog (Shh) pathway. Both presence/increase of primary cilia and their loss have been observed in tumours^73^. The presence/increase of the cilium has been reported both in mesenchymal and epithelial tumours; such as, gastrointestinal stromal tumours^74,75^, giant cell tumours of bone^76^, bladder tumours^77^, meningioma^78^, osteosarcoma^79^, pancreatic adenocarcinomas^80^, lung adenocarcinomas^80^, muscular rabdomiosarcomax^81^, or glioblastoma^44^. The loss of primary cilia has been shown in breast tumours^82,83^, prostate tumours^80,84^, renal tumours^85^, pancreatic adenocarcinomas^86^, colangiocarcinoma^87^, melanoma^88,89^, and glioblastoma cell cultures^90^.

Additionally, the structural dynamism of the primary cilium allows this organelle to appear/disappear at different times or stages of the disease^91,92^. It has been observed that primary cilium can be present both in premalignant and malignant lesions^93–99^. Our results support the presence of primary cilia in low-grade gliomas, although this does not exclude the possibility of their presence in other stages of the tumour, depending on the needs of the cells.

Normal astrocytes show primary cilium to promote cell proliferation and differentiation^91^. In high-grade gliomas, Moser et al*.*^43^ reported an aberrant primary ciliogenesis. However, Sarkisian et al.^44^ showed opposite results. Consistent with these findings, our results suggest that primary cilium is formed in early stages of astrocyte tumour transformation, serving as an antenna that receives signals from the extracellular environment, without displaying any structural alterations.

Exosomes can also be derived from the primary cilium, as shown in previous studies.^56,57,100^ Accordingly, we observed the presence of vesicles in the proximity of ciliary pocket membrane. Masyuk et al*.*^101^ demonstrated for the first time that exosomes can be involved in intercellular communication by interacting with primary cilia, and that ciliary extracellular vesicles contain functional proteins that play a key role in cilia biology. Recent findings have identified that ciliary extracellular vesicles and cytosolic extracellular vesicles have unique and distinct features, highlighting their different properties.^102^ Although we cannot establish the exact origin of the vesicles found close to the ciliary membrane, it is possible that they have an autocrine regulatory effect on the tumour cells themselves via the primary cilium sensing.

## Supplementary Information

Supplementary Information.

## Data Availability

All data generated or analysed during this study are included in this published article and it supplementary information file.
