# Supplementary material for: Spheresomes are the main extracellular vesicles in low-grade gliomas
Source: Sci Rep. 2023 Jul 10;13:11180. doi: 10.1038/s41598-023-38084-y (PMC10333278; doi:10.1038/s41598-023-38084-y)
Supplement: Supplementary file 1 — Supplementary Information. [file 41598_2023_38084_MOESM1_ESM.docx]

**Supplementary Information**

**Extended Materials and Methods**

**Human samples**

Human biopsies were retrieved from surgical resections collected in the Department of Pathology at the University Clinic Hospital of Zaragoza. Five cases of gliomas that fulfilled the histological criteria of low-grade gliomas were examined in this study. All protocols developed were approved by the Human Research Ethics Committee (Comité Ético de Investigación Clínica de Aragón, CEICA) from the Instituto Aragonés de Ciencias de la Salud (permit number: PI16/0324).

**Histological and immunohistochemistry analyses**

Histological samples were processed according to standard histological procedures and stained with hematoxylin and eosin (H&E). Digital images were captured by Olympus BX1 microscope.

Immunohistochemical staining was performed on 2-μm-thick formalin-fixed paraffin-embedded sections using DAKO EnVision® method. Primary antibodies used in these paper were polyclonal rabbit anti-GFAP (1:100, DAKO, Z0334, Glostrup, Denmark), an astroglial marker, and monoclonal mouse anti-Ki-67 (1:100, DAKO, MIB-1, Glostrup, Denmark), a nuclear antigen which recognizes proliferation cells at all stages of the cell cycle. Antibodies were diluted with DAKO diluent (S2022). The tissue sections were deparaffinised in xylene for 20 min and rehydrated in graded ethanol (4 min in 100°, 4 min in 96°, 4 min in 70° and 5 min in distilled water). Prior to all assays, a heat-induced antigen retrieval step was performed using buffer citrate (pH 6, DAKO S2031) by treatment for 5 min in microwave at 800 W and 3 additional minutes at 360 W. After washing twice with PBS for 3 min, endogenous peroxidase was blocked using peroxidase blocking reagent (DAKO, S2001) for 10 min and sections were washed in distilled water and PBS 3 min, twice. Afterwards, sections were incubated with primary antibodies at 4 °C overnight in a humidified chamber. Sections were washed in PBS three times for 5 min and incubated with Polyclonal Rabbit Anti-Goat Immunoglobulins/HRP (1:200, DAKO, P016002-2) for 1 h. To confirm the presence of immunocomplexes, 3,3′-diaminobenzidine as chromogen and hydrogen peroxide as substrate were used. The samples were washed twice in distilled water, contrasted with Mayer’s haematoxylin for 7 min, washed in tap water for 15 min, dehydrated in a graded ethanol (2 min in 70°, 2 min in 96° and 5 min in 100°), cleared in xylene and cover slipped with Eukitt (03989 Sigma-Aldrich; St. Louis, MO, USA). Digital images were captured by Olympus BX1 microscope.

**Immunofluorescence microscopy**

Two-micrometre formalin-fixed paraffin-embedded sections were cut and mounted on glass slides. The sections were deparaffinised in xylene for 20 min and rehydrated in graded ethanol (4 min in 100°, 4 min in 96°, 4 min in 70° and 5 min in distilled water). Tissues were permeabilised with 0.1% Triton X-100 in PBS for 8 min. Afterwards an antigen retrieval step using Tris-buffered saline (TBS, pH 9) was performed at 96 °C for 20 min for Acetylated-tubulin and Pericentrin. Sections were incubated overnight at 4 °C with the following primary antibodies: monoclonal mouse anti-Acetylated- tubulin (1:4000, Sigma Aldrich, T7451; St. Louis, MO, USA) and polyclonal rabbit anti-Pericentrin (1:100, Abcam, ab4448; Cambridge, UK) in a dark humidified chamber. Sections were washed in PBS three times for 5 min and incubated for 1 h at RT in a dark humidified chamber with secondary antibodies: donkey anti-mouse IgG H + L Alexa Fluor 594 (1:1000, ThermoFisher, R37115; Waltham, MA, USA), donkey antirabbit IgG H + L Alexa Fluor 488 (1:1000, ThermoFisher, A-21206; Waltham, MA, USA). After washing in PBS, DAPI (1 μg/ mL, Sigma-Aldrich) was added for 1 min for nuclei counterstaining. Sections were washed in PBS and the slides were covered with fluorescence mounting medium (DAKO, S3023). Samples were visualised with a fluorescence microscope (Olympus BX1 with DP70 Digital Camera System) and analysed with DP Controller Software. Every fluorescent channel was individually photographed and channels were merged using FIJI ImageJ software^54^.

**Electron microscopy**

After tumor resection, samples (about 1–1.5 mm^3^) were washed in phosphate buffer and fixed with 2.5% glutaraldehyde and 2% paraformaldehyde overnight at RT, washed in 0.1 M phosphate buffer for 5 min, post-fixed with 2% osmium, rinsed, dehydrated in graded acetone (30%, 50%, 70% with 2% uranyl acetate, 90%, 100%), cleared in propylene oxide and embedded in araldite (Durcupan, Fluka AG; Buchs SG, Switzerland). Semi-thin sections (1.5 μm) were cut with a diamond knife, lightly stained with 1% toluidine blue and examined by light microscopy (Olympus BX51 microscope, Olympus Imaging Corporation; Tokyo, Japan). Later, ultrathin (0.05 μm) sections were cut, collected on Formvar-coated single-slot grids counterstained with 1% uranyl acetate and Reynold’s lead citrate for 10 min. They were examined under a FEI Tecnai G2 Spirit TEM. The images were captured with Advanced Microscopy Techniques, using a Corp. Charge-Coupled Device imaging system (CCD from Danvers, MA, USA).
